# The relevance of netnography to the harness of Romanian health care electronic word-of-mouth

**Published:** 2014-09-25

**Authors:** R Bratucu, IR Gheorghe, A Radu, VL Purcarea

**Affiliations:** "Carol Davila" University of Medicine and Pharmacy, Bucharest, Romania

**Keywords:** netnography, health care services, online marketing, electronic word-of-mouth

## Abstract

Abstract

Nowadays, consumers use the computer mediated communication to make purchase decisions on a large variety of products and services. Since health care services are archetypal by nature, consumers in this field are one of the most encountered users of electronic word-of-mouth.

The objective of this paper is to explain and support the necessity of adopting a different qualitative method when electronic word of mouth is harnessed on health care dedicated forums, that is, netnography.

## Introduction

Consumers are increasingly using computer medicated communication, such as websites, newsgroups, chat rooms and forums with the purpose of sharing ideas and building communities which would help them make better decisions on product and service choices. This is important especially in high involvement services such as health care, where the internet offers the opportunity to change the method of interaction between both consumer users and service professionals, by providing information resources. 

**Fig. 1:**
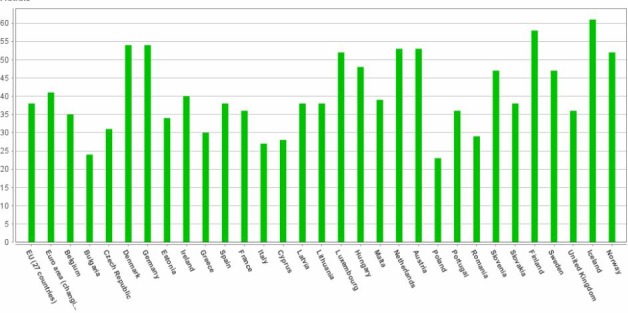
The process of health care seeking information on the Internet in Europe, 2011 [**3**]

 In health care services, patients and potential consumers are increasingly turning to the communications between their known peers in communities, through the internet. Nowadays, such computers mediated communities, called virtual communities, are becoming popular among consumers across different demographic and behavioral backgrounds. As such the internet has been considered a supportive means of search for goal-oriented consumer information and, in health care services, as the harbinger of the consumer behavior movement [**1**]. There took place an upsurge of online health information, when the majority of Internet users have searched for health information using the online environment [**2**]. For instance, in 2011, the European country with the highest percentage of population who looked for online health information was Iceland whereas the country with the lowest percentage was Poland (**Fig. 1**).

**Fig. 2:**
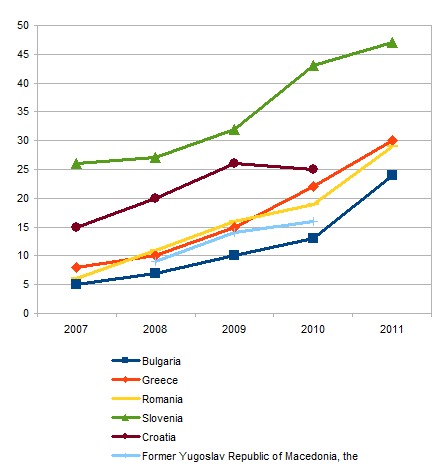
Health Seeking Information on the Internet in South-Eastern Europe, in 2011 [**3**]

 In South-Eastern Europe, the country with the highest rate of online health information search was Slovenia, followed by Greece and Romania (**Fig. 2**).

 Moreover, Internet is considered an active means of finding information on a specific topic. So, the health care consumer implied that the health information is easily organized and accessible on specific topics and themes on health web sites. 

 A sensitive topic is one "that potentially poses for those involved a substantial threat, the emergence of which renders problematic for the researcher and/or the researched the collection, holding and/or dissemination of research data" [**4**]. According to Sieber [**5**], sensitive topics raise wider issues to the ethical, political and legal aspects of research. Moreover, sensitive topics frequently address "some of society’s most pressing social issues and policy questions" [**6**] such as health care issues, and, in addition, Lee [**4**] adds: "Sensitive research is important too precisely because it illuminates the darker corners of society".

 Therefore, the purpose of this paper is to explain and support the necessity of adopting a different qualitative method when electronic word of mouth is harnessed on health care virtual communities, that is, netnography.

 1. Health care services and virtual communities

 Health care services are widely accepted as being designed to be archetypal [**7**]. An important characteristic of this service is the perception that the potential consumer lacks knowledge and expertise in both diagnostic skills and treatment options, while the health care provider stands as the specialist, who has relevant skills and knowledge. However, along with the advancement of technology and the internet and the patients’ access to virtual communities, the professional service encounter could potentially be altered by the consumers’ access to healthcare information and support in the shape of second opinions in written textual formats found on dedicated forums, which in the literature is known as electronic word-of-mouth. 

 Virtual communities are the coffee houses of the online environment where people gather, to talk and listen by reading, to satisfy needs and curiosities, to form or express opinions, to learn, or to build relationships [**8**]. Every user, at a certain period of time, becomes both a consumer of information and a provider. Above all, users who change their positions into members of a virtual community, protect the quality of the community they represent with a full range of methods used to cooperate, share, and exchange information.

 Moreover, literature on virtual communities pays little attention on how they are built form a theoretical perspective. More specifically, not all virtual communities are built and developed under similar objectives. Hagel and Armstrong [**8**] focused on classifying virtual communities according to the needs served as follows:

 - Communities of relationship, where people with similar experiences come together and form meaningful personal relationships.

 - Communities of interest, which aggregate people who share an interest or expertise in a specific topic.

 - Communities of transaction, where information is traded among participants.

 - Communities of fantasy, for exploring new forms of entertainment.

 Bressler [**9**] modified the classification of virtual communities after a business perspective taking into account the motivations of participants:

 - Purpose- where people are trying to achieve the same objective.

 - Practice- where people share the same profession or situation.

 - Circumstance- similar to purpose, but driven by personal experiences rather than professional motivations.

 - Interest- where people share a common passion.

 Nonetheless, virtual communities have also been classified by their focus [**10**] as follows:

 - Basic human needs- interest, relationship, fantasy, transaction.

 - User social structure, or technology-base- e.g., FAQ, expert knowledge, news groups, web site.

 - Motivation- communities of purpose, practice, circumstance, interest.

 These various categorization schemes of virtual communities reveal the following particularities from a consumer perspective: 

 - Sense of belonging to a community; 

 - Bonding;

 - Shared interest among other peers. 

 Specific to health care services management are virtual communities which are especially built and structured around pharmaceutical and health care consumption issues [**11**]. However, there should be made a difference between health care virtual communities and the traditional patient support groups. Laing et al [**12**] argue that virtual communities have lower borders in participation compared to traditional information sources such as patient support groups and, in addition, in virtual communities, time and commitment requirements are lower, and the patient can remain anonymous. Most of times, health care virtual communities offer alternative diagnosis and treatment, mutual support and counseling from other peers. So, health care virtual communities may provide the necessary tools for online information search, self-diagnosis and word of mouth communication, leading to efficient knowledge building and sharing activities. As a result, although they are popularly called "virtual communities"[**13**], the term "virtual", in health care services, might be misleadingly used, suggesting that these communities are less "real" than physical communities [**14**]. Yet as [**11**] pointed out, "these social groups have a "real" existence for their participants, and thus have consequential effects on many aspects of behavior, including consumer behavior". 

 Patient online communities (POCs) have as common purpose the provision of necessary tools to ease the patient’ s process of getting informed as well as self-diagnose or spread efficiently WOM. Even if notions such as "self-help groups" [**15**] or "social support groups" ([**16**], p. 693) are used to define POCs, these communities are, in fact, "communities of unintended interest" [**17**] in which patients post messages when they confront themselves with situations that could change their lives in a short period of time. Although the main objective of POCs is to help patients become more informed as well as improve their capacity of facing difficult facts, Baym [**18**] suggested other sound objectives such as: offering social support, information, influencing public opinions and helping users improve their health care knowledge. Most POCs are considered to be supportive online communities [**19**], offering informational support, nurturing support and instrumental support [**20**]. 

 • The information support refers to data related to treatment or coping with a disease through different action-treatment plans [**21**]. 

 • The nurturing support is expressed through linguistic signs of sympathy and empathy as well as listening [**21**] 

 • The instrumental support encompasses solutions that provide material or/and financial help. 

 2. Electronic word-of-mouth

 Word of mouth is a consumer-generated channel of marketing communication where the sender is independent of an organization, namely, it is a user generated content. Thus, it is perceived to be more reliable, credible and trustworthy by consumers in comparison to organization-planned communications [**22**]. Traditional communications theory considers WOM as having a powerful influence on behavior, especially on consumers’ information search stage, evaluation stage and, on the outcome, the decision making [**23**]. It is the credibility of WOM that, combined with the idea that a receiver will be more involved in a WOM exchange than in an advertisement, lends to higher beliefs and cognitions. Through multiple exchanges, one WOM message might reach and supposing, influence many receivers, similar to electronic word-of-mouth (e-WOM) which is a more modern approach to our era. 

 In the light of the growth rate of internet usage and its role in the field of e-commerce, e-WOM has today a massive impact on the peoples’ behaviors and decisions [**24**]. It can be spread through a variety of electronic means such as forums, email [**25**], online communities or discussion boards [8, 31]. Henning-Thurau et al. [26, p. 39] defined e-WOM as being any "positive or negative statement made by potential, actual or former customers about a product or company, which is made available to a multitude of people and institutions via the Internet". Furthermore, e-WOM is also considered to an asynchronous process which facilitates the "informal communications directed at other consumers about the ownership, usage or characteristics of particular goods or their sellers" [**27**] on a one-to-world platform reaching unlimited users who are sharing experiences [**28**].

 Varadarajan and Yadav [**29**] identified four major changes that have occurred in the online environment since the emergence of e-WOM in the consumer behavior:

 - Access to price and non-price product attributes;

 - Alternative comparisons and evaluations based on the buyer’s considerations;

 - Improved quality of information;

 - Organized and structured information.

 The e-WOM phenomena on the internet has been studied as a result of the psychological motives of the opinion leaders [**26**], whereas, other experts have considered it a process which takes place due to the social ties established between individuals on the internet [**30**]

 3. Netnography

 Online communication between consumers has been studied by using netnography [**31**] in order to understand their attitudes, perceptions and feelings. According to Kozinets [**11**], the internet offers increased opportunities for social group participation, in the shape of virtual communities of consumption so as to assess social power, to unite, and to claim symbols and ways of life that are meaningful to them and, implicitly, to their communities. As such, Kozinets [**11**] defined netnography as "a new qualitative research methodology that adapts ethnographic research techniques to study cultures and communities that are emerging through computer-mediated communications" [**32**]

**Fig. 3:**
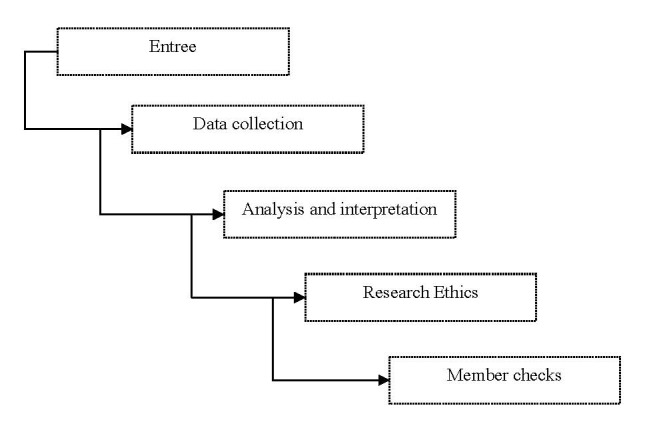
Methodological steps employed by the netnography

 Compared to other methods used in research marketing, netnography is less time consuming, potentially less obtrusive, and less costly. Referring to common ethnographic procedures, Kozinets [**32**, p. 63] recommends the following methodological stages and procedures (**Fig. 3**):

 - Entrée: formulation of research questions and identification of appropriate online forum for study. 

 - Data collection: Direct copy from the computer-mediated communications of online community members and observations of the community and its members, interactions and meanings. 

 - Analysis and interpretation: classification, coding analysis and contextualization of communicative acts

 - Research ethics: (1) The researcher should fully disclose his or her presence, affiliations, and intentions to online community members during any research; (2) the researchers should ensure confidentiality and anonymity of informants; (3) the researchers should seek and incorporate feedback from members of the online community being researched and (4) The researcher should take a cautious position on the private-versus-public medium issue. This procedure requires the researcher to contact community members and to obtain their permission (inform consent) to use any specific postings that are to be directly quoted in the research [**32**, p. 65].

 - Member checks: Presentations of some or all final research report’s findings to the people who have been studied in order to solicit their comments. 

## Conclusion

CMC interaction provides some anonymity as opposed to face-to-face communication, which "allows a person to discuss fears, ask factual questions and discuss common experience to reduce isolation" [**33**] and at the same time "to help each other cope with shared problems [**34**]. Although social interaction through CMC resembles face-to-face interactions, the anonymity characteristic can produce the outcome of intimacy and closeness because it passes the issue of stigma, which may cause ridicule and rejection among peers [**35**]. CMC increases the number of users as a person may overcome both geographic and time constraints [**35**]. 

 Chuang and Yang [**36**] conducted a research which supports previous findings that users engage in online health support communities with the interest of finding peers with similar experiences and to seek social support. Finding other patients with similar issues is important because listening to others’ stories one person can uncover hidden facets of the situation he or she is facing. Specialists in order to penetrate the barriers of intimacy without intruding have to find the proper method which is netnography.
